# Predicting Grape Sugar Content under Quality Attributes Using Normalized Difference Vegetation Index Data and Automated Machine Learning

**DOI:** 10.3390/s22093249

**Published:** 2022-04-23

**Authors:** Aikaterini Kasimati, Borja Espejo-García, Nicoleta Darra, Spyros Fountas

**Affiliations:** Laboratory of Agricultural Machinery, Department of Natural Resources Management and Agricultural Engineering, Agricultural University of Athens, 75 Iera Odos Str., 11855 Athens, Greece; borjaeg@aua.gr (B.E.-G.); nicoletadarra@aua.gr (N.D.); sfountas@aua.gr (S.F.)

**Keywords:** NDVI, AutoML, Bayesian optimization, ensemble methods, correlation, quality prediction, sugars

## Abstract

Wine grapes need frequent monitoring to achieve high yields and quality. Non-destructive methods, such as proximal and remote sensing, are commonly used to estimate crop yield and quality characteristics, and spectral vegetation indices (VIs) are often used to present site-specific information. Analysis of laboratory samples is the most popular method for determining the quality characteristics of grapes, although it is time-consuming and expensive. In recent years, several machine learning-based methods have been developed to predict crop quality. Although these techniques require the extensive involvement of experts, automated machine learning (AutoML) offers the possibility to improve this task, saving time and resources. In this paper, we propose an innovative approach for robust prediction of grape quality attributes by combining open-source AutoML techniques and Normalized Difference Vegetation Index (NDVI) data for vineyards obtained from four different platforms-two proximal vehicle-mounted canopy reflectance sensors, orthomosaics from UAV images and Sentinel-2 remote sensing imagery-during the 2019 and 2020 growing seasons. We investigated AutoML, extending our earlier work on manually fine-tuned machine learning methods. Results of the two approaches using Ordinary Least Square (OLS), Theil-Sen and Huber regression models and tree-based methods were compared. Support Vector Machines (SVMs) and Automatic Relevance Determination (ARD) were included in the analysis and different combinations of sensors and data collected over two growing seasons were investigated. Results showed promising performance of Unmanned Aerial Vehicle (UAV) and Spectrosense+ GPS data in predicting grape sugars, especially in mid to late season with full canopy growth. Regression models with both manually fine-tuned ML (R² = 0.61) and AutoML (R² = 0.65) provided similar results, with the latter slightly improved for both 2019 and 2020. When combining multiple sensors and growth stages per year, the coefficient of determination R² improved even more averaging 0.66 for the best-fitting regressions. Also, when considering combinations of sensors and growth stages across both cropping seasons, UAV and Spectrosense+ GPS, as well as Véraison and Flowering, each had the highest average R² values. These performances are consistent with previous work on machine learning algorithms that were manually fine-tuned. These results suggest that AutoML has greater long-term performance potential. To increase the efficiency of crop quality prediction, a balance must be struck between manual expert work and AutoML.

## 1. Introduction

Precision viticulture is a method of managing vineyard variability by using spatiotemporal data and observations to maximize a vineyard’s oenological potential. New vineyard management technologies have made it possible to increase production efficiency and quality while reducing environmental impacts [1,2]. In situ estimation of productivity variables is time-consuming and unreliable. It involves visual inspection of vines and grapes for number, color, shape, size and other information based on the grower’s own expertise, similar to fruit trees [3]. Grape quality, which refers to the degree of excellence of grape composition characteristics [4], is often described in terms of sugar and titratable acidity at harvest. Currently, laboratory tests on samples are the most common method for determining grape quality characteristics. This involves analyzing representative samples of berries or grapes using standard wet chemical techniques on extracts obtained at regular intervals to determine the maturity of grapes in a vineyard block [5]. Although this method is very accurate, its main disadvantage is that it can be time-consuming and costly [6].

The use of remote sensing has become widespread in viticulture, especially for monitoring grape growth and estimating grape quality and yield. Canopy response and Normalized Difference Vegetation Index (NDVI) are commonly used for spatial decision making in vineyards [7]. Proximal, aerial, and satellite sensors and platforms are used in various configurations to record canopy characteristics [8,9].

In the past, research has been conducted to evaluate the quality and yield of grapes using vegetation indexes (VIs) derived from various sensors. A common approach is to conduct statistical and regression analyses, including descriptive statistics, Pearson correlation to determine the spatial relationship between canopy NDVI and crop quality and yield [10,11,12], and linear and multivariate regression models to determine field-wide production [10]. In recent years, computer power has increased significantly, allowing more sophisticated machine learning approaches to predict crop yield and quality [13,14,15,16]. Tree-based ensemble methods, such as boosted regression trees and random forests, as well as computer vision [17,18], have also been used to test more advanced yield estimation methods alongside linear regression models [19,20].

Machine learning-based data analysis is actively used as a fast and one of the most effective methods to predict yield and quality. Although the application of machine learning in agriculture is new, it is currently being used at a rapid pace [21]. However, the widespread use of machine learning techniques remains a challenge, as their successful application is not effortless. These techniques still rely heavily on specialized human resources [22]. They usually require the extensive involvement of experts working iteratively to develop the most appropriate machine learning pipeline, as the highly complex agricultural environment requires complex algorithms for data analysis and a thorough understanding of mathematics, coding, and extensive experience in selecting model architecture [23,24]. Moreover, any machine learning-based solution faces the “no free lunch” theorem [25], which means that no algorithm is going to be the best solution for every dataset; and, thus, neither the most powerful algorithm is going to work for all yield/grape quality prediction problems. Therefore, it would be ideal if non-experts could automatically build a machine learning pipeline tailored to each scenario.

Automated machine learning (AutoML) offers the opportunity to improve this task and save time and human-resources by automating the time-consuming, iterative tasks of machine learning model development, including model selection and hyperparameter tuning. AutoML systems are meta-level machine learning algorithms that find the optimal machine learning pipeline topologies based on previous machine learning solutions [23,24]. These systems automatically evaluate alternative pipeline designs and attempt to iteratively improve performance for a given task and dataset [26]. Additionally, experienced engineers can benefit from AutoML solutions that result in better models being deployed in less time. At the same time, they can provide new users with an understanding of how such models work, what data they require, and how they can be applied to typical agricultural problems. However, one of the drawbacks of AutoML systems is that they require a significant amount of computing power.

While previous research has explored various correlation and regression models between VIs and crop production, as well as machine learning techniques for estimating grape yield and quality, as described above, AutoML has not been widely explored. In the agricultural field, the use of AutoML technique has only been recorded for time series processing and proximal and satellite imagery analysis [27,28] and weed identification [29]. In this paper, we propose an innovative approach for robust prediction of grape quality attributes by combining open-source AutoML techniques and NDVI data for vineyards obtained with non-destructive methods from four different platforms-two proximal vehicle-mounted canopy reflectance sensors, orthomosaics from UAV images and Sentinel-2 remote sensing imagery- at different growth stages during the 2019 and 2020 growing seasons.

## 2. Materials and Methods

### 2.1. Solution Workflow

**Figure 1:**
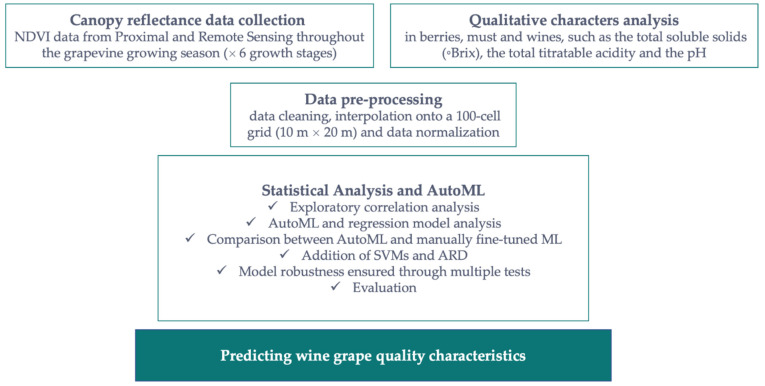
Workflow to test the suitability of automated machine learning for predicting grape sugars using NDVI data from proximal and remote sensing.

In this paper, we propose an alternative approach for robust prediction of grape quality attributes by combining open-source AutoML techniques and NDVI data collected at different growth stages with non-destructive methods such as remote sensing currently used in precision viticulture (Figure 1). We investigated AutoML, extending our previous work on manually fine-tuned machine learning methods [16]. A comparison was made between the two methods, manually fine-tuned machine learning and AutoML. Support Vector Machines (SVMs) and Automatic Relevance Determination (ARD) were included in the analysis and different configurations, such as using different combinations of sensors and data collected over two growing seasons, were explored.

The study included several sets of high-resolution multispectral data obtained from four sources, including two vehicle-mounted sensors to detect plant reflectance, data collected by an UAV, and archived Sentinel-2 imagery to determine the characteristics of grapevine canopies at different growth stages. Several techniques were used to preprocess the data, including data quality assessment, interpolation of the data onto a 100-cell grid (10 m × 20 m), and normalization of the data. The transformed data set was then processed and applied to statistical analysis and AutoML. These algorithms were first trained on the available data distribution and then validated and tested with linear and non-linear regression models, including Ordinary Least Square (OLS), Theil-Sen and Huber regression models, ensemble methods based on decision trees, Support Vector Machines (SVMs), and Automatic Relevance Determination (ARD).

### 2.2. Study Area

A commercial vineyard on the Palivos Estate in Nemea, Greece (37.8032°, 22.69412°, WGS84) served as the field site for the study. The vineyard, planted with Vitis vinifera L. cv. ‘Agiorgitiko’ for wine production, is located on a steep slope and the experimental area selected for data collection was approximately 2 ha. Wine grapes were trained to a vertical shoot positioned, cane pruned double Guyot training/trellis system, with northeast-southwest row orientation.

### 2.3. Canopy Reflectance Data Collection

**Figure 2:**
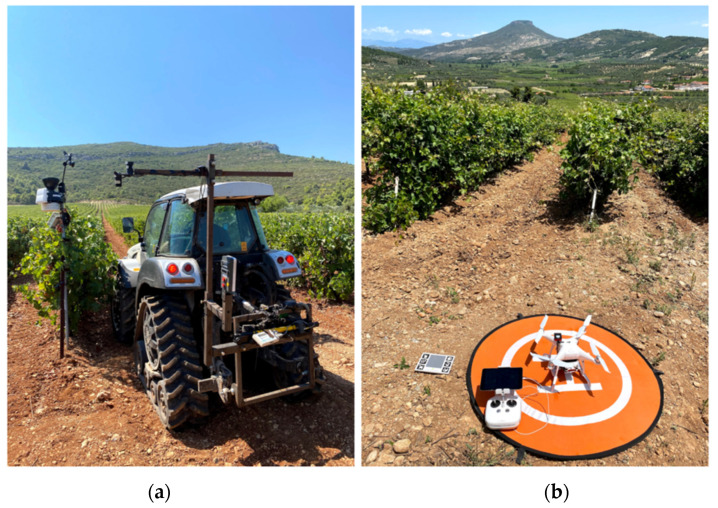
Grapevine canopy properties were estimated using (**a**) two vehicle-mounted crop reflectance sensors, (**b**) UAV-acquired data and Sentinel-2 archived imagery.

To assess NDVI at different vine phenological growth stages, canopy reflectance was measured six times per growing season in 2019 and 2020, beginning in late May and ending at harvest in early September. Two vehicle-mounted proximal sensors were used to assess plant vigor at these six berry growth stages, namely (i) shoots, (ii) flowering, (iii) setting, (iv) pea-sized berries, (v) véraison, (vi) harvest, while a UAV and Sentinel-2 satellite imagery were used to assess plant vigor through remote sensing. A CropCircle, active proximal canopy sensor (ACS-470, Holland Scientific Inc., Lincoln, NE, USA) and a Spectrosense+ passive GPS sensor (Skye Instruments Ltd., Landrindod Wells, UK) were mounted on a tractor at the correct height from the soil surface and horizontally at an appropriate distance from the vines during each growth stage to record proximal reflectance measurements from the side and top of the canopy, respectively (Figure 2a). A Garmin GPS16X HVS (Garmin, Olathe, Kansas, USA) and the built-in Spectrosense + GPS were used to georeference all recorded data. Aerial data were collected on the same dates as the proximal measurements using a Phantom 4 Pro drone (Dà-Jing Innovations, Shenzhen, Guangdong, China) equipped with a multispectral Parrot Sequoia+ camera (Parrot SA, Paris, France) and GPS so that all photos could be geotagged (Figure 2b). Cloud-free and atmospherically corrected Sentinel-2 satellite images, 2A products with a spatial resolution of 10 m (S2 spectral bands operate at the different spatial resolution of 10 m -4 bands, B2, B3, B4, and B8), were acquired via the ESA portal, the official Copernicus Open Access Hub (www.scihub.copernicus.eu, accessed on 2 October 2020), providing reflectance values at the bottom of the atmosphere in cartographic geometry for the data closest to the proximal and UAV surveys. This generally occurred within 2 days during mid-and late-season surveys, but was as much as 9 days after preseason ground-based observations due to heavy cloud cover in the closest digestion dates (Table 1).

### 2.4. Data Preparation

**Figure 3:**
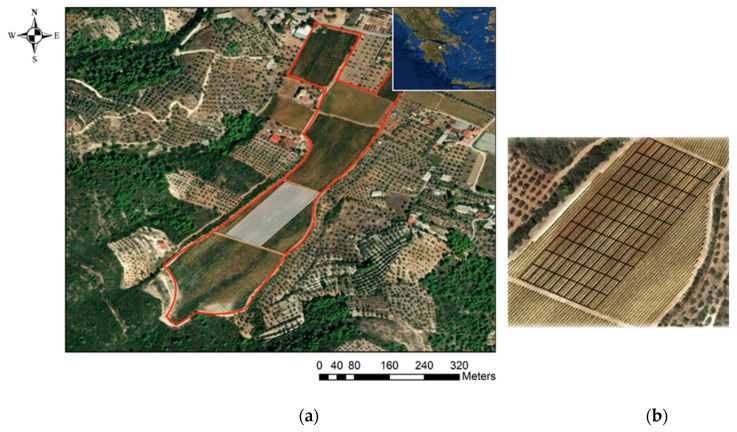
A satellite image showing (**a**) the national and local position of the commercial vineyard and (**b**) the 100-cell grid constructed parallel to the trellis lines.

All proximal canopy reflectance data were projected (UTM Zone 34N), cleaned by deleting data points outside field boundaries, and interpolated according to Taylor et al. (2007) [30]. ArcMap v10.3 (ESRI, Redlands, CA, USA) was used to scale up the interpolated data to 10 m × 20 m cells. The Zonal Statistics tool was used to display the index values per block based on the average of the pixels located in the same area. In this way, 100 plots were created throughout the study region, resulting in NDVI map time series with a spatial resolution of 10 m × 20 m that were aligned parallel to the trellis lines. Similarly, Pix4D software (Pix4D S.A., Prilly, Switzerland) was used to integrate data acquired by drone, and the resulting NDVI orthomosaic was fitted to vineyard boundaries. The data were then upscaled to the same 100 plots using an averaging method. Prior to upscaling the data to the 10 m × 20 m plots, a spatial correction of the NDVI values within the plots was applied to the Sentinel-2 imagery that followed the boundaries of the experimental field(Figure 3). The “Shift (Data Management)” command was used, which moves the grid to a new geographic position based on x and y offset values.

### 2.5. Qualitative Characters Analysis

Grapes were harvested by hand at the end of each growing season in mid-September. A standard grid of 100 cells (10 m × 20 m) covering the entire area was configured to facilitate field sampling and to evaluate grape yield and quality. Total yield was calculated by counting the total number of crates filled with grapes per cell and multiplying this number by the average crate weight of harvested grapes [11,12]. By randomly selecting fifty berries from each vineyard cell, the qualitative characteristics of the grapes were analyzed. Total soluble solids (◦Brix), total titratable acidity and pH of the berries, must and wines were determined. Qualitative analysis of the common vineyard quality indicators, total soluble solids in must, total titratable acidity and pH, was performed according to Stavrakaki et al. (2018) [31] at the Laboratory of Viticulture, Agricultural University of Athens.

### 2.6. Statistical Analysis

A preliminary descriptive statistical analysis was performed to investigate the effectiveness of proximal and remote sensing in predicting grape quality. In the exploratory correlation analysis, the Pearson correlation matrix was used to evaluate the relationships between NDVI data from all four proximal and remote sensors and grape quality attributes.

### 2.7. Architecture of the Solution

**Figure 4:**
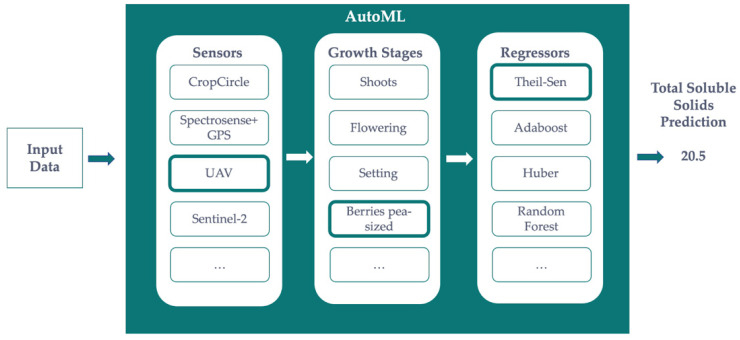
Architecture of the AutoML-based solution proposed in this research.

Figure 4 shows how the AutoML-based solution is envisioned for the prediction of any precision agriculture metrics, such as yield or sugar ◦Brix content. Given some measurements from different sensors on different growth stages, the Bayesian optimization method that runs under the AutoML solution will find the best combination of algorithms and hyper-parameters. It is important to note that every machine learning algorithm has a different set of hyper-parameters to fine-tune. This means that there is no a priori knowledge of the best fit, and they are not optimized during the learning process. For instance, the number of trees for Random Forests and AdaBoost, the split criterion (e.g.: Gini, Entropy, etc.) for all tree-based methods, and the sensitivity against outliers of robust linear regression methods, such as Theil-Sen or Huber. The AutoML will find the best combination of these hyper-parameters before deciding which is the best machine learning method to use. The use of ensembles of fine-tuned pipelines is out of the scope of this paper. As an example, Figure 4 depicts a combination of UAV data on Berries pea-sized and Theil-Sen regression as the best pipeline for predicting the Sugar ◦Brix Content. This pipeline optimization is done manually when not using AutoML.

### 2.8. Regression Methods and AutoML Setup

Although AutoML could use an endless bunch of machine learning algorithms, in this work, AutoML was investigated to extend our previous work on manually fine-tuned machine learning methods [16]. Linear and nonlinear regression algorithms were used, including Ordinary Least Square, Theil-Sen, and Huber regression models, as well as decision trees, depending on which initial model was developed.

- **Ordinary Least Square (OLS)**: The most common estimation method for computing linear regression models, which can be found in related works such as, Prasetyo et al. (2018) [32].
- **Theil-Sen Estimator Method**: It is the most popular non-parametric technique for estimating a linear trend, and makes no assumption about the underlying distribution of the input data [33].
- **Huber Regression**: It is aware of the possibility of outliers in a dataset and assigns them less weight than other samples, unlike Theil-Sen, which ignores them [34].
- **Decision Trees**: This method uses a non-parametric learning approach. Its main advantage is that it is easy to interpret. Unless the model is too complicated, it can be visualized to better understand why the classifier made a particular decision.

To improve the predictive power of our model, this study also evaluated several ensemble methods based on decision trees such as AdaBoosting, Random Forests, and Extra Trees. These combine the predictions of multiple machine learning algorithms to make more accurate predictions than the individual models. All of these ensemble methods start with a decision tree and then use boosting or bootstrap aggregation to reduce its variance and bias (bagging).

- **AdaBoost**: The AdaBoost algorithm (adaptive boosting) uses an ensemble learning technique known as boosting, in which a decision tree is retrained several times, with greater emphasis on data samples where regression is imprecise [35].
- **Random Forest**: A supervised learning approach in which the ensemble learning method is used for regression. This combines numerous decision tree regressors into a single model trained on many data samples collected on the input feature (in this case, NDVI) using the bootstrap sampling method [36].
- **Extremely Randomized Trees**: Extra Trees is similar to Random Forest in that it combines predictions from many decision trees, but instead of bootstrap sampling, it uses the entire original input sample [37].

Although tree-based approaches offer a way to go beyond parametric model constraints, they have the disadvantage of being computationally more expensive than traditional OLS. However, they should be a good technique to address the regression modeling problem if the performance differences are large enough.

The results of the two approaches, manually fine-tuned machine learning and AutoML, using the above methods were compared. In addition, Support Vector Machines (SVM) and Automatic Relevance Determination (ARD) were included in the analysis and different combinations of sensors and data collected over two growing seasons were examined.

- **Support Vector Machines**: It is one of the most robust prediction methods. The (non-linear) model produced by this algorithm depends only on a subset of the training data because the cost function does not take into account any training data close to the model predictions [38].
- **Automatic Relevance Determination**: It is the regularization of the solution space using a parameterized, data-dependent priority distribution that effectively removes redundant or superfluous features [39].

Moreover, since according to Gupta (2018) [40], it is possible to make better predictions when only a few variables are considered rather than all attributes, all NDVI measurements were studied individually and using combinations of two. Consequently, some automation is lost, but a richer analysis for research and knowledge dissemination purposes could be done.

### 2.9. Evaluation Methodology

The coefficient of determination (R²) and root mean square error (RMSE) were used to evaluate the predictive accuracy and determine the performance of the models for the best sensor or season [12,41,42,43]. In addition, a 5-fold cross-validation was performed for each regression model to check its generalization ability and ensure its robustness. The experiments were also repeated 10 times to ensure that the final results were as accurate as possible.

### 2.10. Software and Hardware

The main software package used in this study was the Auto-Sklearn machine learning library (version 0.14.2). Auto-Sklearn is an open-source library that uses the Scikit-Learn machine learning library (version 0.24.2) for data transformations and machine learning to perform AutoML [23,44]. Its deployment is very similar to that of the data scientist, which increases the reliability of the process. It finds a powerful model pipeline for a given set of features by using a Bayesian optimization search approach. The experiments were all performed on Ubuntu 18.04 as the operating system.

## 3. Results

### 3.1. Exploratory Correlation Analysis

In an exploratory correlation analysis, the Pearson correlation matrix was used to evaluate the correlations between the NDVI data of all four proximal and remote sensors and the grape quality indices. Absolute correlations between NDVI data from all four proximal and remote sensors and total soluble solids, sugar content measured in ◦Brix (|*r*| > 0.50), were typically good for both 2019 and 2020, with the signal stabilizing in the middle and end of the growing season. During the pea-sized berries and ripening season in 2019, the Spectrosense + GPS data showed the highest correlation (|*r*| = 0.74). (i.e., mid and late season at full canopy growth). The UAV data had the strongest correlations (|*r*| = 0.79) during the same growth stages in 2020. All Sentinel-2 NDVI variables had weak associations (0.29 < |*r*| < 0.57) compared to total soluble solids. Total titratable acidity and pH, the other two important grape quality criteria, were not associated with NDVI data at any vine phenological growth stage. The full results were presented and discussed in detail in our earlier paper [16].

### 3.2. Regression Analysis

#### 3.2.1. Comparing Manually Fine-Tuned ML and AutoML

The manually fine-tuned regression models between the NDVI data of all four sensors and the total soluble solids showed different degrees of accuracy depending on the fitted model, the sensor used and the growth stage evaluated [16]. The best-fitting regressions, both linear and nonlinear, were observed mainly for UAV and Spectrosense + GPS data, in mid-late season with full canopy growth, during pea-sized berries and at the Véraison growth stage. The best-fitting model was found to estimate total soluble solids during Véraison, with a coefficient of determination R² in the range of (0.38 < R² < 0.61) for both 2019 and 2020. The highest coefficient of determination for the regression models (R² = 0.61) was observed for the UAV-derived NDVI data for 2020, while for 2019 the canopy reflectance data derived from the CropCircle and Spectrosense + GPS proximal sensors appeared to have better performance in predicting grape quality traits. When using AutoML, the R² values improved slightly to (0.49 < R² < 0.65) for both 2019 and 2020. Similar to the manually fine-tuned ML, AutoML gave the maximum coefficient of determination of R² = 0.65 for the UAV-derived NDVI data for 2020 during Véraison and R² = 0.57 for the Spectrosense + GPS data for 2019. Finally, the RMSE is generally reduced when using AutoML. The selected best results of the AutoML regression algorithms compared to the manually fine-tuned algorithms for the evaluation of grape quality attributes are shown in Table 2.

#### 3.2.2. Combination of Sensors and Growth Stages

Using AutoML, several combinations of sensors and growth stages per year were investigated to evaluate their performance in assessing grape quality attributes. The coefficient of determination R² of 0.57 to 0.66 for the two years was significantly better than when only one sensor/growth stage was considered. For 2019, the best fitting regressions (R² = 0.58) were observed for Spectrosense + GPS NDVI data in combination with the other sensors (CropCircle, UAV and Sentinel-2) mainly during Véraison. On the other hand, for 2020, the best fitting regressions (R² = 0.66) for UAV NDVI data in combination with the other sensors (CropCircle, Spectrosense + GPS and Sentinel-2) were observed mainly during Véraison, but also during Flowering. The selected best R² per year for combined sensors and growth stages using AutoML to evaluate their performance in assessing grape quality traits are shown in the following table (Table 3).

The best sensor-based R² and growth stage-based R² per year for combined sensors and growth stages using AutoML to evaluate their performance in assessing grape quality traits are also presented below in Table 4 and Table 5. Considering the ‘sensor’ as the common denominator, the coefficient of determination R² between 0.44 and 0.54 for 2019 and 0.31 and 0.64 for 2020. The Spectrosense + GPS (0.52 < R² < 0.54), followed by the CropCircle (R² = 0.48), appears to perform better in predicting TSS in 2019, especially at the Véraison, Flowering and Berry pea-sized growth stages, while the UAV (0.46 < R² < 0.47) and Sentinel-2 (R² = 0.44) sensors follow. Keeping the sensor as a constant, the change in prediction performance within the growth stages is very small. For 2020, the UAV and Spectrosense + GPS perform best in the evaluation of grape quality attributes (R² > 0.5) among different combinations of growth stages. The NDVI data derived from CropCircle do not seem to be able to provide good insight into grape sugar estimation, giving low R² values (0.31 < R² < 0.34), while the Sentinel-2 images do not provide any information for assessing grape quality in 2020.

Taking ‘growth stage’ as the common denominator, the coefficient of determination R² varies between 0.38 and 0.58 for 2019 and between 0.52 and 0.66 for 2020. Véraison (R² < 0.57) is the growth stage that gives the best prediction for TSS in both years followed by the Berries pea-sized (0.39 < R² < 0.47) and Flowering (R² = 0.58) in 2019 and 2020, respectively, regardless of the sensing system used. Holding the growth stage constant, the change in predictive performance between the different sensors is very small or even zero.

#### 3.2.3. Combinations over the Two Growing Seasons, 2019 and 2020

**Figure 5:**
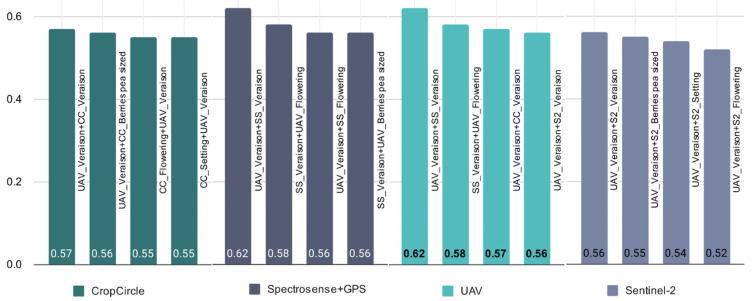
Given a specific sensor (CropCircle, Spectrosense + GPS, UAV, Sentinel-2), selected best performed R^2^ for the combination of sensors and growth stages over the two growing seasons (2019 and 2020), using AutoML to assess their performance in evaluating grape quality attributes (legend as for Table 2).

The next step was to investigate the predictive power of different combinations of sensors and growth stages over the two growing seasons 2019 and 2020. Considering both years and different growth stages, the UAV and Spectrosense + GPS have the highest average R² values of 0.55 and 0.53, respectively (Table 6). The sensor systems CropCicle and Sentinel-2 seem to perform weaker in the evaluation of the grape quality attributes with R² values between 0.24 and 0.36. Figure 5 shows the best R² value for combined sensors and growth stages over the two growing seasons for a given sensor, which also demonstrates the superiority of UAV and Spectrosense + GPS in predicting grape quality.

**Figure 6:**
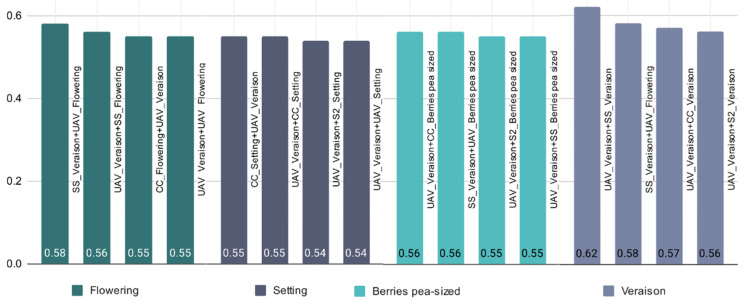
Given a growth stage (Flowering, Setting, Berries pea-sized, Véraison) selected best performed R^2^ for the combination of sensors and growth stages over the two growing seasons (2019 and 2020), using AutoML to assess their performance in evaluating grape quality attributes (legend as for Table 2).

Looking at both years and sensors, the Véraison and Flowering growth stages appear to have the highest average R² values of 0.62 and 0.48, respectively (Table 7). The Setting and Berry pea-sized growth stages seem to perform worse in the evaluation of the grape quality attributes with R² values between 0.30 and 0.36. Figure 6 shows the best R² values for combined sensors and growth stages across the two growing seasons when considering a specific growth stage. This also shows that collecting NDVI data during Véraison and Flowering is useful for better prediction of grape quality.

Using AutoML, a number of regression algorithms, namely OLS, Theil-Sen and Huber regression models, tree-based methods SVMs and ARD, were explored in the analysis. Table 8 shows them in alphabetical order along with the corresponding R² values and ranking of the best solutions. ARD, Huber regression and SVM have the highest R² values and at the same time the highest positions in the ranking, while Random Forest had the lowest R² values and yet was ranked as the second best solution. This is due to the fact that Random Forest acts as an “all-rounder” algorithm that gives decent results for all sensors and growth stages. In general, the tree ensembles performed poorly overall, which may well be related to the low predictive power of decision trees in this problem (R² = 0.45).

## 4. Discussion

This work extended and enriched our earlier research on manually fine-tuned machine learning methods [16]. An innovative approach for robust prediction of grape quality attributes was proposed by combining open-source AutoML techniques and vineyard NDVI data collected at different growth stages with non-destructive methods such as remote sensing. While previous research has explored various correlation and regression models between VIs and crop production, as well as machine learning techniques for estimating grape yield and quality, AutoML has not been extensively explored, as described above. In the agricultural field, the use of AutoML technique has only been recorded for time series processing and analysis of proximal and satellite imagery [27,28] and weed identification [29]. The results of the manually fine-tuned ML and AutoML using OLS, Theil-Sen and Huber regression models and tree-based methods were compared. SVMs and ARD were included in the analysis and different combinations of sensors and data collected over two growing seasons were investigated. In addition, a 5-fold cross-validation was performed for each regression model to check its generalization ability and ensure its robustness. The experiments were also repeated 10 times to ensure that the final results were as accurate as possible.

Several research studies have been conducted, especially in the last few decades, looking at the use of proximal and remote sensors in viticulture. Bramley et al. [45], Primicerio et al. [46], Taskos et al. [47], Reynolds et al. [48], and Darra et al. [12] used VIs from proximal and remote sensing imagery to assess vineyard condition and its relationship with yield variability, while Sozzi et al. [49] and Matese et al. [50] used VIs from S2 and UAV sensors to monitor vineyards. Arnó et al. [51] and Henry et al. [52] used different proximal sensors to assess vineyard characteristics, while other researchers, such as Xue and Su [53], used different remote sensors (hyperspectral or thermal) for the same reason. Multi-annual measurements during different growth stages, as selected in the present study, seem to be a reliable source of information to draw reliable conclusions about plant development, as investigated and highlighted in several other previous studies, e.g., by Lamb et al. [54], Kazmierski et al. [55], Anastasiou et al. [42]. The correlation analysis for each development stage separately aimed to distinguish the most important period for plant development and its correlation with production.

The canopy reflectance data recorded by all four sensors, i.e., the pure NDVI of the vines extracted from two proximal sensors, a CropCirle and a Spectrosense + GPS, as well as the ‘mixed pixel’ of the UAV and Sentinel-2 images, showed an increasing correlation with the total soluble solids as the season, according to the exploratory correlation analysis. NDVI data collected with the UAV, Spectrosense + GPS, and the CropCircle during the Berries pea-sized and Véraison stages, in the middle-late season with full canopy growth, showed the highest correlations with sugar content in both years. Similar results, showing that NDVI at late developmental stages has good correlations with crop yield and attributes of TSS, were also found by other researchers in Greek viticultural systems [11,56,57]. Relationships between mid- to late-season NDVI and yield were also found by Garcia-Estevez et al. (2017) in Spain [58] (Véraison NDVI) and Sun et al. (2017) (pre-harvest NDVI) in California [10]. The lower correlation coefficients collected with Sentinel-2 and analyzed with an overhead ‘mixed pixel’ technique showed that the predictions for grape quality were less reliable. The difference between the strong correlations of the Sentinel NDVI layers with the other sensors in 2019 and the weaker correlations of these satellite layers with all other sensors in 2020 was a troubling result of the analysis. It also indicated a divergence between the satellite platform and the terrestrial and UAV observations in close proximity, even when these higher resolution data were upscaled and correlations were performed at a similar scale to the satellite imagery. The reason for the lower satellite imagery performance in 2020 is unknown, and there was no clear evidence of system failure. In contrast, the two other primary quality parameters for wine grapes, total titratable acidity and pH, showed no correlation with the NDVI data at any crop stage.

The regression models between NDVI data from all four proximal and remote sensors and total soluble solids gave similar results with both manually fine-tuned ML and AutoML, with the latter slightly improved for both 2019 and 2020. These results are in line with Bhatnagar and Gohain (2020), who used decision tree and random forest-based machine learning algorithms to estimate crop yields by comparing their values with NDVI data, resulting to an R² = 0.67 [59]. More accurate predictions of grape quality were obtained when NDVI data were collected close to harvest date, although promising results were also obtained for early season, as also noted in the study by Ballesteros et al. (2020) [14]. Different degrees of accuracy were observed depending on the sensor used and the growth stage assessed. The UAV and Spectrosense + GPS data were found to be more accurate in predicting sugar content from all grape quality attributes, especially in mid-late season at full canopy growth, Berries pea-sized and Véraison growth stages, achieving a coefficient of determination of R² = 0.65 for the UAV-derived NDVI data for 2020 during Véraison and R² = 0.57 for the Spectrosense + GPS data for 2019. This is due to the fact that NDVI data from both proximal and remote sensing show strong similarities between NDVI values obtained from similar sensors in both statistical and production contexts, but diverge with increasing distance between platforms, resulting in NDVI maps that are not the same when converted to production decisions [60].

When combining multiple sensors and growth stages per year, the coefficient of determination R² improved. For 2019, the best-fitting regressions for Spectrosense + GPS NDVI data in combination with the other sensors (CropCircle, UAV and Sentinel-2) were mainly observed during Véraison. On the other hand, for 2020, the best fitting regressions for UAV NDVI data in combination with the other sensors (CropCircle, Spectrosense + GPS and Sentinel-2) were observed mainly during Véraison, but also during Flowering. The situation is similar when looking at the combinations of sensors and growth stages across the two growing seasons 2019 and 2020: the sensors UAV and Spectrosense + GPS as well as Véraison and Flowering each have the highest average R² values. The sensor systems CropCicle and Sentinel-2 seem to be weaker in the evaluation of grape quality traits together with the Setting and Berry pea-sized growth stages. This means that if one has to choose a sensor to invest in to collect NDVI data to predict grape quality traits, the best options are a UAV or a Spectrosense + GPS. Similarly, if someone is able to collect NDVI data only twice during the growing season, the best times during the growing season are the Véraison and Flowering growth stages. Finally, a number of regression algorithms were tested using AutoML. ARD, Huber Regression and SVM had the highest R² values and at the same time the highest positions in the ranking, while Random Forest had the lowest R² values and yet was ranked as the second best solution. This is due to the fact that Random Forest acts as an “all-rounder” algorithm that gives decent results for all sensors and growth stages. It is important to note, however, that deciding which specific regressors to use is not a critical issue when using AutoML. On the other hand, since resources are always scarce, knowing which algorithms are the most promising and focusing on them could save computational and thus economic resources. From a viticultural perspective, the improved predictive power of AutoML offers the opportunity to reduce the cost of data collection, either by making the most appropriate investment in sensor systems and/or by identifying the best combination of sensors and vine growth stage to perform measurements. In the long term, it is proposed to use two sensors for more robust prediction of grape quality characteristics, as not all combinations work.

Therefore, better performance has been achieved by using AutoML, which frees the machine learning user from selecting algorithms and tuning hyperparameters, and takes advantage of Bayesian optimisation and meta-learning. The AutoML system of choice was Auto-sklearn because of its excellent results and deployment capabilities. It showed improved performance over the state of the art for various combinations within the dataset. Since Auto-sklearn is based on the algorithms of ML implemented in the Scikit-learn library, its application would be very similar to that of the data scientist, increasing the reliability of the process. One of the implementations that have made a great advance in automating modeling is the use of meta-learning techniques as implemented in Auto-sklearn. This replaces the “intuition at first sight” of the experts with learning from the obvious features of the input data. On the other hand, it is important to note that the performances came from different machine learning algorithms/pipelines. For example, in the ten experiments where UAV_Véraison was used as NDVI input, five used SVM, three used Huber regression and two used OLS. Unlike our previous research where both NDVI input and regression methods were discussed equally, in this research the regression methods are secondary and subordinate to AutoML consistency to achieve the best performance. According to the results presented and considering the No Free Lunch theorem, it can be discussed that it would be more informative to discuss methods that automatically fine-tune different ML pipelines where the specific regressor (e.g., SVM, Adaboost, etc.) is only a hyperparameter, rather than emphasizing the superiority of a specific machine learning method.

For some specific sensors and growth stages, the performances achieved were high. For example, UAV_Véraison + SS _Véraison with an R² between 0.58 for 2019 and 0.66 for 2020 with an RMSE of 1.08–1.16. One could debate whether this is the minimum error that can be achieved. As the use of ensemble construction is outside the scope of the AutoML pipeline studied in this paper, it cannot be claimed that the reported results are the best that can be achieved with AutoML technology. For example, using bagging, boosting or stacking as ensemble frameworks that reuse the best performing pipelines could improve performance and should be explored in future work. Finally, it could be discussed that even the most sophisticated AutoML method could fail in finding a predictive relationship if some specific NDVI measurements are used that are obviously unrelated and could be used as part of an over-fitted model.

## 5. Conclusions

This paper investigates the application of open-source AutoML and multi-platform multi-temporal NDVI data for the evaluation of quality attributes of grapes. This extends and strengthens the results of our previous research on manually fine-tuned machine learning methods [16]. Descriptive statistical analysis showed that the NDVI data from overhead systems (UAV and Spectrosense + GPS) and CropCircle, during the Berries pea-sized and Véraison stage, in the middle-late season with full canopy growth, had the strongest correlations with sugar content for both years, while the Sentinel-2 images were less reliable for predicting grape quality attributes. The Sentinel-2 data showed significantly weaker correlations with TSS, indicating a problem with their quality, especially for 2020. This is an indication that the use of proximal sensing can provide earlier and more accurate estimates of important quality attributes without being compromised by soil background effects and lower resolution, as is the case with satellite-based NDVI data. Regression models gave similar results with both manually fine-tuned ML and AutoML, with the latter slightly improved for both 2019 and 2020. When combining multiple sensors and growth stages per year, the coefficient of determination R² improved even more. Similarly, when looking at the combinations of sensors and growth stages across both 2019 and 2020 cropping seasons, the overhead sensors, as well as Véraison and Flowering, each have the highest average R² values. A number of regression algorithms have been tested with AutoML and produced better results than manually fine-tuned ML. These performances come from different machine learning algorithms/pipelines, thus increasing predictive power and providing a more reliable and sustainable solution that can be used in the long term. This research will be extended by evaluating bagging, boosting or stacking as ensemble frameworks that reuse the best performing pipelines and investigating whether they could lead to better performance. Finally, given the perennial nature of grapevines and the various environmental and endogenous factors that determine quality, seasonal calibration for quality prediction should be considered in future research.

## Figures and Tables

**Table 1:** Grapevine seasonal EL growth stages of proximal and remote sensing data acquisition. Dates reported in Table 1 relative to both seasons (2019 and 2020).

Dates	EL No-Stage	Description
15 May–30 May	12-Shoots	5 leaves separated; shoots about 10 cm long; inflorescence clear
1 June–20 June	23-Flowering	16–20 leaves separated; 50% caps off
21 June–20 July	27-Setting	Young berries enlarging, bunch at right angles to stem
21 July–15 August	31-Berries pea-sized	About 7 mm in diameter
16 August–10 September	35-Véraison	Berries begin to color and increase in size
11 September–20 September	38-Harvest	Berries ready for harvest

**Table 2:** Comparison of selected best R2 values with (a) Manually fine-tuned ML (adapted from Kasimati et al., 2021a) and (b) AutoML to evaluate their performance in assessing the wine grapes quality characteristics (CC, CropCircle; SS, Spectrosense + GPS; UAV; and S2, Sentinel-2).

	(a) Manually Fine-Tuned ML			(b) AutoML		
	Sensor_Growth Stage	R² (avg)	RMSE	Sensor_Growth Stage	R² (avg)	RMSE
**2019**	SS_Véraison	0.51 ± 0.09	1.45 ± 0.19	SS_Véraison	0.57 ± 0.05	1.14 ± 0.29
	CC_Véraison	0.42 ± 0.10	1.67 ± 0.35	UAV_Véraison	0.52 ± 0.04	1.22 ± 0.25
	SS_Berries pea-sized	0.41 ± 0.11	1.71 ± 0.24	S2_Berries pea sized	0.49 ± 0.06	1.30 ± 0.48
	UAV_Véraison	0.38 ± 0.10	1.95 ± 0.55	CC_Véraison	0.49 ± 0.08	1.25 ± 0.26
**2020**	UAV_Véraison	0.61 ± 0.03	1.37 ± 0.19	UAV_Véraison	0.65 ± 0.04	1.22 ± 0.36
	UAV_Berries pea-sized	0.57 ± 0.04	1.55 ± 0.32	UAV_Flowering	0.59 ± 0.05	1.30 ± 0.41
	UAV_Flowering	0.56 ± 0.06	1.75 ± 0.19	UAV_Berries pea sized	0.59 ± 0.05	1.31 ± 0.32
	SS_Setting	0.44 ± 0.0	1.73 ± 0.23	SS_Setting	0.54 ± 0.03	1.44 ± 0.53

**Table 3:** Selected best performed R^2^ per year for combined sensors and growth stages using AutoML to evaluate their performance in assessing the wine grapes quality characteristics (legend as for Table 2).

	Combined Sensor_Growth Stage	R² (avg)	RMSE
**2019**	SS_Véraison + UAV_Véraison	0.58 ± 0.06	1.08 ± 0.33
	SS_Véraison + UAV_Setting	0.57 ± 0.06	1.08 ± 0.34
	SS_Véraison + CC_Véraison	0.57 ± 0.08	1.09 ± 0.3
	SS_Véraison + S2_Véraison	0.57 ± 0.07	1.10 ± 0.35
**2020**	UAV_Véraison + SS_Véraison	0.66 ± 0.07	1.16 ± 0.36
	UAV_Véraison + S2_Véraison	0.66 ± 0.07	1.17 ± 0.35
	UAV_Véraison + S2_Flowering	0.66 ± 0.06	1.17 ± 0.34
	CC_Flowering + UAV_Véraison	0.65 ± 0.07	1.19 ± 0.38

**Table 4:** Selected best performed sensor-based R^2^ per year for combined sensors and growth stages using AutoML to evaluate their performance in assessing the wine grapes quality characteristics (legend as for Table 2).

	Sensor-Based Combined Sensor_Growth Stages		R² (avg)	RMSE
**2019**	SS_Véraison +	SS_Flowering	0.54 ± 0.06	1.13 ± 0.29
		SS_Véraison	0.53 ± 0.08	1.14 ± 0.32
		SS_Berries pea sized	0.52 ± 0.08	1.13 ± 0.21
	CC_Véraison +	CC_Véraison	0.48 ± 0.15	1.20 ± 0.34
	UAV_Véraison +	UAV_Flowering	0.47 ± 0.12	1.21 ± 0.29
		UAV_Berries pea sized	0.46 ± 0.14	1.22 ± 0.19
		UAV_Setting	0.46 ± 0.11	1.24 ± 0.33
	S2_Berries pea sized +	S2_Flowering	0.44 ± 0.22	1.24 ± 0.38
**2020**	UAV_Véraison +	UAV_Flowering	0.64 ± 0.08	1.20 ± 0.36
		UAV_Berries pea sized	0.64 ± 0.07	1.20 ± 0.39
		UAV_Setting	0.62 ± 0.11	1.22 ± 0.42
	SS_Setting +	SS_Flowering	0.55 ± 0.07	1.35 ± 0.37
		SS_Véraison	0.53 ± 0.07	1.36 ± 0.38
		SS_Harvest	0.51 ± 0.06	1.39 ± 0.32
	CC_Setting +	CC_Berries pea sized	0.34 ± 0.1	1.62 ± 0.58
		CC_Harvest	0.32 ± 0.13	1.66 ± 0.82
		CC_Véraison	0.31 ± 0.15	1.65 ± 0.66

**Table 5:** Selected best performed growth stage-based R^2^ per year for combined sensors and growth stages using AutoML to evaluate their performance in assessing the wine grapes quality characteristics (legend as for Table 2).

	Growth Stage-Based Combined Sensors_Growth Stage		R² (avg)	RMSE
**2019**	SS_Véraison +	UAV_Véraison	0.58 ± 0.06	1.08 ± 0.33
		CC_Véraison	0.57 ± 0.08	1.09 ± 0.3
		S2_Véraison	0.57 ± 0.07	1.10 ± 0.35
	S2_Berries pea sized +	UAV_Berries pea sized	0.47 ± 0.01	1.22 ± 0.31
		SS_Berries pea sized	0.42 ± 0.24	1.25 ± 0.29
		CC_Berries pea sized	0.39 ± 0.18	1.31 ± 0.43
	UAV_Flowering +	SS_Flowering	0.38 ± 0.17	1.32 ± 0.48
		CC_Flowering	0.38 ± 0.11	1.33 ± 0.48
**2020**	UAV_Véraison +	SS_Véraison	0.66 ± 0.07	1.16 ± 0.36
		S2_Véraison	0.66 ± 0.07	1.17 ± 0.35
		CC_Véraison	0.64 ± 0.07	1.20 ± 0.37
	UAV_Flowering +	S2_Flowering	0.58 ± 0.08	1.29 ± 0.37
		UAV_Flowering	0.58 ± 0.07	1.29 ± 0.35
		CC_Flowering	0.58 ± 0.07	1.29 ± 0.34
	UAV_Berries pea sized +	CC_Berries pea sized	0.55 ± 0.08	1.33 ± 0.44
		SS_Berries pea sized	0.55 ± 0.08	1.34 ± 0.43
	SS_Setting +	UAV_Setting	0.52 ± 0.09	1.38 ± 0.39
		S2_Setting	0.52 ± 0.07	1.39 ± 0.35

**Table 6:** Given a specific sensor, the best combination of growth stages and the corresponding best R2 over the two growing seasons (2019 and 2020), using AutoML to assess their performance in evaluating grape quality attributes (legend as for Table 2).

Sensor	Combined Growth Stages	R² (avg)	RMSE
CropCircle	Setting + Véraison	0.36 ± 0.18	1.47 ± 0.5
Spectrosense + GPS	Véraison + Flowering	0.53 ± 0.1	1.26 ± 0.3
UAV	Véraison + Flowering	0.55 ± 0.06	1.20 ± 0.31
Sentinel-2	Flowering + Berries pea sized	0.24 ± 0.16	1.63 ± 0.55

**Table 7:** Given a specific growth stage, the best combination of sensors and the corresponding best R2 over the two growing seasons (2019 and 2020), using AutoML to assess their performance in evaluating grape quality attributes (legend as for Table 2).

Sensor	Combined Growth Stages	R² (avg)	RMSE
Flowering	CC + UAV	0.48 ± 0.04	1.31 ± 0.41
Setting	CC + UAV	0.36 ± 0.14	1.46 ± 0.48
Berries pea-sized	SS + S2	0.30 ± 0.19	1.55 ± 0.47
Véraison	UAV + SS	0.62 ± 0.05	1.13 ± 0.34

**Table 8:** List of the algorithms used in AutoML to assess their performance in evaluating grape quality attributes, the corresponding R^2^ values and the ranking of each algorithm.

Algorithm	R² (avg)	Best Solution (Rank)
Adaboost	0.44 ± 0.09	7
ARD	0.53 ± 0.09	3
Decision Tree	0.45 ± 0.11	8
Extra Trees	0.43 ± 0.08	9
Huber Regression	0.52 ± 0.12	4
SVM	0.52 ± 0.12	1
Random Forest	0.41 ± 0.09	2
OLS	0.51 ± 0.09	6
Theil-Sen Regression	0.51 ± 0.12	5

## Data Availability

The data presented in this study are available on request from the corresponding author. The data are not publicly available due to privacy of the data collection location.
